# Changes in cardiovascular health and physical functioning in non‐hospitalized, adult COVID‐19 patients after 3 years of follow‐up

**DOI:** 10.14814/phy2.70868

**Published:** 2026-04-17

**Authors:** Janneke I. A. Vloet, Esmée A. Bakker, Koen M. van der Sluijs, Guilherme F. Speretta, Armando van der Horst, Thijs M. H. Eijsvogels, Dick H. J. Thijssen

**Affiliations:** ^1^ Department of Medical BioSciences Radboud University Medical Center Nijmegen The Netherlands; ^2^ Department of Primary and Community Care Radboud University Medical Center Nijmegen The Netherlands; ^3^ Department of Physiological Sciences, Biological Sciences Center Federal University of Santa Catarina Florianopólis Brazil; ^4^ Laboratory of Clinical Chemistry and Haematology Jeroen Bosch Hospital ‘s‐Hertogenbosch The Netherlands; ^5^ Research Institute for Sports and Exercise Sciences Liverpool John Moores University Liverpool UK

**Keywords:** cardiovascular health, incident infection, long‐term effects, non‐severe COVID‐19, physical functioning

## Abstract

Long‐term cardiovascular consequences of COVID‐19 remain unclear, particularly in non‐hospitalized individuals. We investigated longitudinal changes (~3 years) in cardiovascular risk factors, cardiac biomarkers, physical functioning, and activity in non‐hospitalized COVID‐19 individuals versus age‐ and sex‐matched controls. Assessments were performed in 2021 (~6 months post‐infection) and repeated 3 years after initial SARS‐CoV‐2 infection among 101 non‐hospitalized COVID‐19 participants and 101 healthy age‐ and sex‐matched controls. Multiple imputation was adopted to adjust for missing data due to drop‐out. Based on the absence (−) or presence (+) of infections during follow‐up, four groups were created: COVID‐19/− (*n* = 45), COVID‐19/+ (*n* = 56), Control/− (*n* = 29), and Control/+ (*n* = 72). Additionally, sex‐stratified analyses were performed. No changes were found in parameters related to cardiovascular risk factors, cardiac biomarkers, physical functioning and physical activity across the 3‐year follow‐up, showing no time × group effects for any of the outcomes (all *p*‐values >0.05). Sex‐stratified analyses and complete‐case sensitivity analysis reinforced our results. Overall, we found no changes in cardiovascular risk factors, cardiac biomarkers, physical functioning and activity across a 3‐year follow‐up in non‐hospitalized COVID‐19 individuals compared to healthy controls, independent of infections during follow‐up. This suggests that non‐hospitalized COVID‐19 did not induce long‐term deleterious changes in cardiovascular health or physical characteristics.

## 1 INTRODUCTION

Coronavirus Disease 2019 (COVID‐19) has been associated with a range of cardiovascular complications during and immediately following infection, particularly among patients with severe acute infections who were hospitalized (Clerkin et al., 2020; Long et al., 2020). As the COVID‐19 pandemic transitioned into an endemic phase, attention has shifted towards the potential longer‐term consequences of a SARS‐CoV‐2 infection. Even mild infections, that is, representing the majority of infections globally, trigger acute subclinical inflammation and/or subtle alterations in cardiovascular function (Puntmann et al., 2022; Todorovic, 2022). Therefore, it is important to understand the possible persistent effects of COVID‐19.

Epidemiological data from the early pandemic suggest that hospitalized and non‐hospitalized individuals with COVID‐19 had an elevated 1‐year risk for incident cardiovascular disease (e.g., cerebrovascular disease, dysrhythmias, inflammatory heart disease, and thrombotic disorders) and mortality compared to individuals free of a COVID‐19 infection (Wan et al., 2023; Xie et al., 2022). Studies also examined the impact of COVID‐19 on cardiovascular biomarkers and risk factors but reported conflicting results. For example, we (van der Sluijs et al., 2023) and others (Kilinc et al., 2025) found no differences in cardiac biomarker concentrations and/or physical functioning between non‐hospitalized COVID‐19 patients and non‐COVID‐19 controls 6 months post‐infection. In contrast, a recent study found mild‐to‐severe COVID‐19 to be associated with increased arterial stiffness 1‐year following infection, a finding primarily observed in women (Bruno et al., 2025), suggesting potential sex‐related differences in longitudinal changes. These discrepant findings may be related to the strong variation in follow‐up (3–12 months), disease severity, and population health status, which complicates comparison across studies. Importantly, the impact of COVID‐19 is scarcely studied beyond 12 months following infection. This limits our current understanding of the potential impact of COVID‐19 on cardiovascular health.

Therefore, we examined longitudinal changes in cardiovascular risk factors, cardiac biomarkers, physical functioning, and physical activity in non‐hospitalized individuals with COVID‐19 across a 3‐year follow‐up period. For this purpose, we re‐invited 101 individuals with a COVID‐19 infection (in 2020/21) and 101 age‐ and sex‐matched controls at 3 years following their initial SARS‐CoV‐2 infection to repeat assessments of their cardiovascular health and functional status (van der Sluijs et al., 2023). Additionally, we examined whether longitudinal changes were sex specific. We hypothesized that, in line with our observations at 6 months post‐infection, no longitudinal changes or differences between non‐hospitalized individuals with COVID‐19 and their controls in cardiovascular risk factors, cardiac biomarkers, physical functioning, and physical activity were present in this relatively healthy, non‐hospitalized cohort.

## 2 MATERIALS AND METHODS

### 2.1 Study design and population

This is a longitudinal follow‐up study of a sub‐cohort of the Nijmegen Exercise Study (van der Sluijs, Bakker, et al., 2025), a large cohort study including relatively healthy and physically active participants, which originally tested 101 adults ~6 months after their initial SARS‐CoV‐2 infection in 2021 who were matched for age and sex with non‐infected controls (van der Sluijs et al., 2023). An inclusion criterion for the COVID‐19 group was evidence of a positive polymerase chain reaction (PCR) test for SARS‐CoV‐2, whereas an exclusion criterion was hospitalization due to the SARS‐CoV‐2 infection. Age and sex‐matched controls were only included if they never had signs, symptoms, or suspicions of a SARS‐CoV‐2 infection nor a lifetime positive test of any sort for SARS‐CoV‐2 before study participation. Dutch language proficiency and residency were inclusion criteria for both groups. The follow‐up assessments were performed in 2024, ~36 months following the initial COVID‐19 infection and ~30 months following the original evaluation in 2021. Based on the initial presence of COVID‐19 (COVID‐19 versus Control), but also the absence (−) or presence (+) of incident infection during follow‐up, subjects were allocated to 4 groups: COVID‐19/−, COVID‐19/+, Control/−, and Control/+. Participants were invited by e‐mail for a single visit to our research center (Radboud university medical center, Nijmegen, The Netherlands). All study procedures were kept similar between the two assessments. Randomization was not applicable to the current and previous measurements of this observational cohort study. The study procedures were approved by the local ethics committee Oost‐Nederland (2023‐16721) and the study was conducted in accordance with the Declaration of Helsinki. All participants provided written informed consent prior to participation in the study.

### 2.2 Sample size

As our study has an explorative character, no formal sample size calculation was performed a priori. In the primary study, the Cohen's D effect size for the outcome measure mean arterial pressure was 5, with an alpha of 0.05 and beta of 0.2 (power of 0.8). According to G*power (Version 3.1), the (a posteriori) estimated sample size needed was 79 participants in each study arm, so 158 in total. As groups were further subdivided based on COVID‐19 status in the present study, these analyses should be interpreted as exploratory.

### 2.3 Measurements

#### 2.3.1 COVID‐19 characteristics

Upon follow‐up, participants completed a questionnaire to assess demographics, general and cardiovascular health status, as well as characteristics related to COVID‐19, including information on the number of incident infections, vaccination status, and hospitalization due to infection.

#### 2.3.2 Cardiovascular risk factors

Height (m) and weight (kg; measured with Seca, Hamburg, Germany) were assessed, and body mass index (BMI, kg/m^2^) was calculated. Noninvasive left brachial blood pressure (mmHg) and heart rate (beats/min) were measured twice after 5 min of rest in a supine position using a semi‐automated blood pressure device (M3, OMRON, Healthcare Europe, Hoofddorp, The Netherlands). The average values of the two measurements were used for analysis. Venous blood was drawn from the antecubital vein (8.5 mL, BD Vacutainer SST II Advance) and coagulated for 45–60 min before being centrifuged at 3000 revolutions/min for 10 min at 4°C. Serum was then transferred to 2‐mL microtubes and stored at −80°C until analysis. The following biomarkers were analyzed: total cholesterol, high‐density lipoprotein (HDL), low‐density lipoprotein (LDL), triglycerides, glucose hexokinase, insulin, creatinine, and C‐reactive protein (CRP). Biochemical analyses were performed batchwise on Atellica and IMMULITE 2000 analyzers (Siemens Healthcare Diagnostics, Tarrytown, NY) at the Jeroen Bosch Hospital (‘s‐Hertogenbosch, The Netherlands). Smoking behavior was inquired via questionnaires.

#### 2.3.3 Cardiac blood biomarkers

From the venous blood samples, high‐sensitive cardiac troponin I (hs‐cTnI) and amino‐terminal pro‐B‐type natriuretic peptide (NT‐proBNP) were analyzed. Biochemical analyses were performed batchwise on Atellica and IMMULITE 2000 analyzers (Siemens Healthcare Diagnostics, Tarrytown, NY) at the Jeroen Bosch Hospital (‘s‐Hertogenbosch, The Netherlands).

#### 2.3.4 Physical functioning and physical activity

Peak handgrip strength (kg) of the nondominant hand was measured three times, separated by 1‐min intervals using the Jamar hydraulic, according to Webb et al. (1989). The single highest value was used in the analysis. Gait speed (km/h) was assessed twice over a 4‐m stretch with 2‐m acceleration and deceleration zones on either side to ensure a stable pace. The fastest try was used in the analysis. This method follows the 4‐m gait speed protocol, as described in the Short Physical Performance Battery (Webb et al., 1989). Ambulant physical activity monitoring (8 days, 24 h) was performed with an ActivPAL3 micro‐accelerometer (PAL Technologies, Glasgow, UK). During this period, participants were requested to keep a sleep/wake diary to enable automated analysis of 24‐h activities. A minimum of 4 valid days was used as an inclusion criterion for analysis. Data were extracted via PALbatch (PAL software Suite, version 8, PAL Technologies) and analyzed using a modified version of the script by Winkler et al. (2016), Bakker et al. (2021) in SAS (Statistical Analysis System, RRID: SCR_008567, version 9.4; SAS Inst., Cary, NC) to compute daily light intensity (LIPA) and moderate‐to‐vigorous physical activity (MVPA) duration (min/day), sleeping and sitting time (h/day), and step count (steps/day).

Assessors were not blinded to group status. All measurements were conducted by trained staff using standardized procedures identical to the 2021 assessment to ensure consistency and minimize interrater variability.

### 2.4 Statistical analyses

All parameters were tested for normality by inspecting Q‐Q plots. Normally distributed data were reported as mean (SD), while non‐normally distributed data were reported as median [Q1–Q3] for continuous data. Categorical variables were reported as numbers (%). Missing data were imputed with multiple imputations by chained equations with predictive mean matching, since *n* = 75 participants had missing data of at least one of the follow‐up measurements (~37% missing at follow‐up). After checking for patterns of missing data, the assumption of “missing at random” was followed. All mentioned variables present from the assessment in 2021 and 2024 were used to predict missing values in 5 imputed datasets with 20 iterations. Healthy convergence, imputed distribution, and plausibility were verified.

To compare differences between groups over time, two‐way repeated measures analysis of Variance (ANOVA) was performed, where Control/− served as a reference group. Given two repeated measurements, no additional covariance structure was required. Level of education, age, and sex were considered as confounders in the analyses. Based on findings of recent research, stratified analyses were performed for males and females to explore a potential sex difference (Bruno et al., 2025). Sensitivity analyses were performed using only the complete cases available within the dataset, as well as sensitivity analyses to assess potential differences between participants who participated in the follow‐up measurements and those who did not respond to the invitation. *p*‐values <0.05 were considered significant. *p*‐values of time, group, and interaction between time and group were presented. Statistical analyses were performed in RStudio (2023.12.1 + 402) using packages mice (Van Buuren & Groothuis‐Oudshoorn, 2011) and ggplot2 (Wickham, 2011).

## 3 RESULTS

Of the 202 participants originally enrolled in the study (van der Sluijs et al., 2023), 128 positively responded to the invitation for a follow‐up assessment. Participants had a median age of 58 years [54, 65] at baseline, with 118 (58%) male and 84 (42%) female participants. No outliers were excluded. After multiple imputation by chained equations with predictive mean matching was performed, pooled estimates indicated 45 participants were in the COVID‐19/− group, 56 in the COVID‐19/+ group, 29 in the Control/− group, and 72 in the Control/+ group. All groups showed vaccination rates of 85% or higher at 3‐year follow‐up (Table 1). No significant differences were observed in participant characteristics, cardiovascular risk factors, cardiac biomarkers, physical functioning, and physical activity between groups, except for a higher handgrip strength in the COVID‐19/+ group (47 [34, 53] kg) when compared to the Control/+ group (Table 1).

**TABLE 1:** Population characteristics of the study population across the different groups according to the pooled estimates. For exploring differences between groups for numeric variables (Median [IQR]), a Kruskal–Wallis test is performed. The chi‐squared test or Fisher's exact test is used for categorical variables (*n* (%)).

	COVID‐19/− (*n* = 45)	COVID‐19/+ (*n* = 56)	Control/− (*n* = 29)	Control/+ (*n* = 72)	*p*‐value
Age (years)	60 [54, 67]	59 [55, 65]	60 [56, 64]	57 [53, 64]	0.38
Sex (male, %)	25 (57%)	34 (61%)	18 (70%)	40 (56%)	0.57
Alcohol status (current, %)	38 (86%)	50 (90%)	24 (85%)	65 (91%)	0.28
Smoking status (never, %)	23 (48%)	38 (68%)	16 (55%)	43 (60%)	0.42
Vaccination status (vaccinated in 2024, %)	39 (85.2%)	52 (92.6%)	28 (95.2%)	62 (88.4%)	0.29
Cardiovascular risk factors					
BMI (kg/m^2^)	23.8 [22.2, 25.8]	24.4 [22.3, 25.9]	25.1 [23.3, 26.3]	24.6 [22.8, 26.9]	0.41
MAP (mmHg)	97 [93, 109]	97 [92, 105]	101 [95, 108]	99 [92, 108]	0.78
HR (beats/min)	60 [53, 65]	56 [51, 61]	57 [51, 64]	57 [53, 64]	0.35
Total cholesterol (mmol/L)	5.3 [4.6, 6.2]	5.2 [4.9, 5.8]	5.2 [4.8, 5.7]	5.0 [4.6, 5.8]	0.54
LDL (mmol/L)	3.1 [2.4, 4.0]	3.2 [2.6, 3.5]	3.1 [2.7, 3.7]	3.0 [2.5, 3.6]	0.87
HDL (mmol/L)	1.6 [1.3, 1.8]	1.5 [1.2, 1.8]	1.5 [1.2, 1.7]	1.5 [1.3, 1.8]	0.46
Triglycerides (mmol/L)	1.0 [0.8, 1.4]	1.1 [0.9, 1.4]	1.2 [0.9, 1.6]	1.0 [0.8, 1.3]	0.38
Insulin (mIU/mL)	4.2 [2.2, 7.7]	4.2 [2.1, 8.1]	3.9 [2.1, 5.5]	3.5 [2.0, 5.8]	0.42
Glucose (mmol/L)	5.1 [4.8, 5.4]	5.0 [4.8, 5.3]	5.0 [4.8, 5.2]	4.9 [4.7, 5.2]	0.34
Creatinine (μmol/L)	79.5 [71.0, 89.0]	83.8 [71.2, 90.0]	81.2 [75.1, 92.1]	79.7 [69.7, 87.2]	0.45
CRP (mg/L)	4.0 [4.0, 4.0]	4.0 [4.0, 4.0]	4.0 [4.0, 4.0]	4.0 [4.0, 4.0]	0.48
Cardiac biomarkers					
NT‐proBNP (pg/mL)	8.6 [5.4, 12.8]	8.5 [5.3, 14.3]	7.9 [5.0, 12.4]	6.6 [4.0, 10.7]	0.42
Hs‐cTnI (ng/L)	4.2 [2.5, 7.1]	4.4 [2.7, 10.3]	4.0 [2.6, 5.8]	3.3 [2.5, 7.5]	0.19
Physical functioning					
Handgrip strength (kg)	41 [32, 52]	47 [34, 53]	42 [35, 49]	37 [30, 48]	0.018
4‐m walking speed (km/h)	5.5 [5.1, 6.0]	5.5 [5.1, 6.3]	5.8 [5.1, 6.3]	5.8 [5.3, 6.3]	0.44
24‐h Physical activity behavior					
Sitting time (h/day)	9.0 [8.1, 10.2]	9.3 [8.2, 10.2]	9.4 [8.8, 10.3]	9.2 [8.3, 10.4]	0.75
MVPA (min/day)	102 [87, 121]	105 [79, 129]	116 [92, 138]	108 [86, 132]	0.44
LIPA (min/day)	284 [222, 324]	273 [224, 317]	253 [196, 327]	243 [206, 308]	0.30
Step count (steps/day)	6577 [5612, 8221]	6732 [5101, 8249]	7164 [5765, 8597]	7018 [5707, 8525]	0.61
Sleeping time (h/day)	8.6 [7.6, 9.7]	8.8 [7.8, 9.2]	7.8 [7.1, 8.8]	8.3 [7.3, 9.6]	0.15

Abbreviations: BMI, Body Mass Index; CRP, C‐Reactive Protein; HDL, high‐density lipoprotein; HR, heart rate; Hs‐cTnI, high‐sensitivity cardiac Troponin I; LDL, low‐density lipoprotein; LIPA, light intensity physical activity; MAP, mean arterial pressure; MVPA, moderate‐to‐vigorous physical activity; NT‐proBNP, N‐terminal pro Brain Natriuretic Peptide; SB, sedentary behavior.

### 3.1 Cardiovascular risk factors, cardiac biomarkers and physical functioning and activity

**FIGURE 1:**
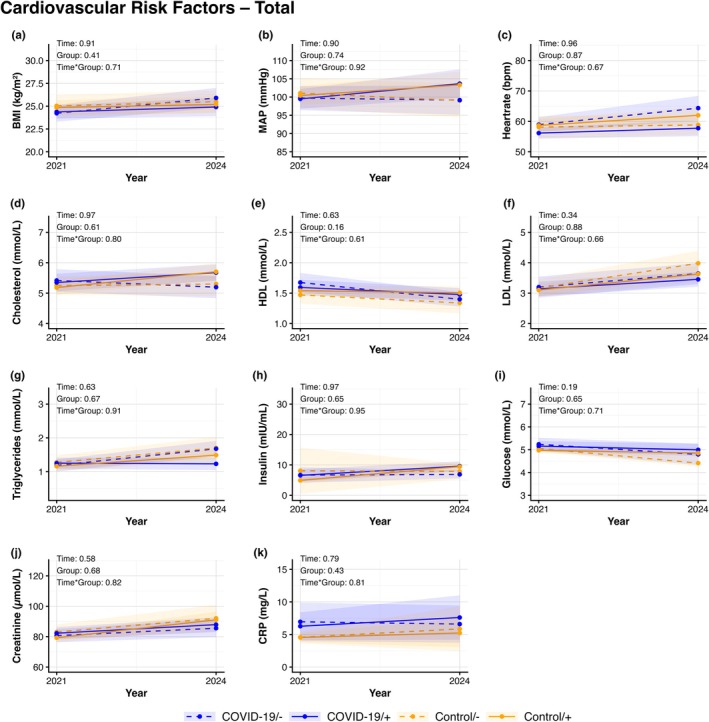
Lines represent data after multiple imputation for cardiovascular risk factors Body Mass Index (BMI, a), mean arterial pressure (MAP, b), Heartrate (HR, c), total cholesterol (d), HDL cholesterol (e), LDL cholesterol (f), triglycerides (g), Insulin (h), glucose Hexikinase (i), creatinine (j), and CRP (k) which are presented at 6‐months and 3‐years follow‐up following initial COVID‐19 infection (blue) and their age‐ and sex‐matched controls (orange), with both groups divided into those with re‐infection (+, solid lines) or free of re‐infection during follow‐up (−, dashed lines). Lines representing mean and 95% CI for the initial (2021) and follow‐up assessment (2024) per group. *p*‐values represent the outcomes of a two‐way ANOVA reflecting the effects of time, group and time × group‐interaction of the outcome.

**FIGURE 2:**
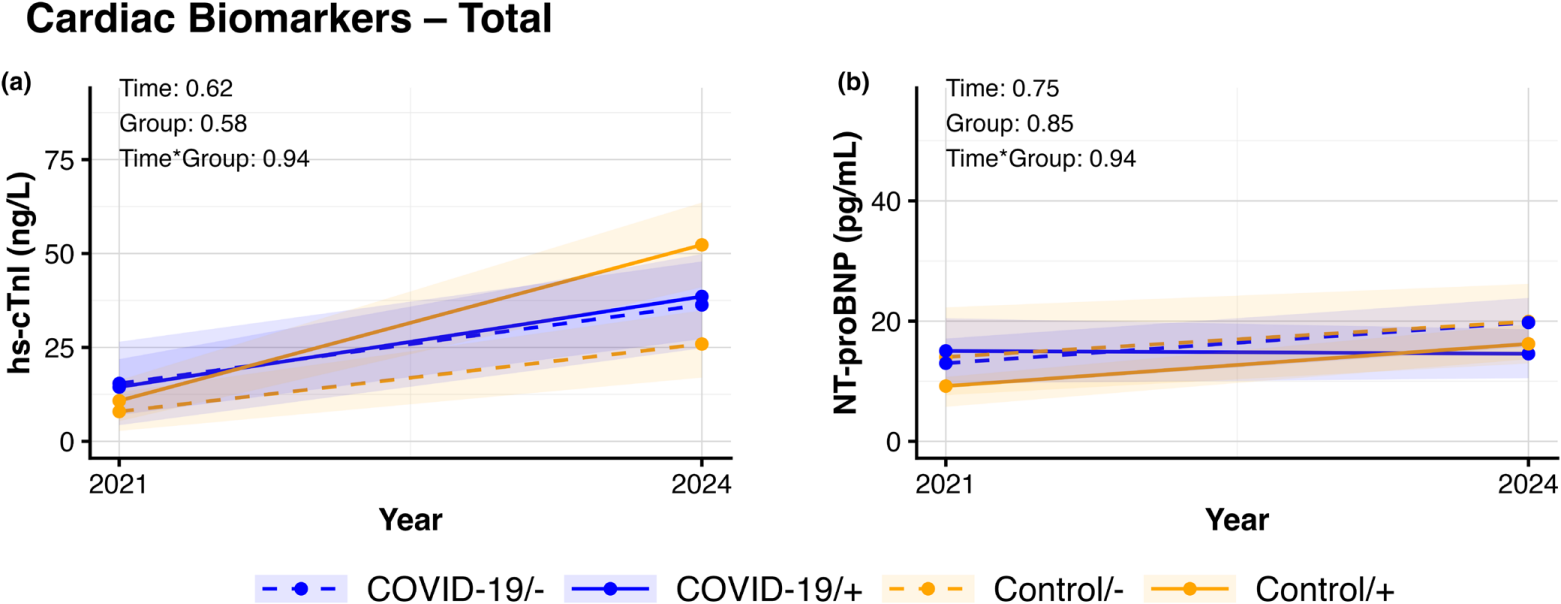
Lines represent data after multiple imputation for cardiac biomarkers hs‐cTnI (a) and NT‐proBNP (b) which are presented at 6‐months and 3‐years follow‐up following initial COVID‐19 infection (blue) and their age‐ and sex‐matched controls (orange), with both groups divided into those with re‐infection (+, solid lines) or free of re‐infection during follow‐up (−, dashed lines). Lines representing mean and 95% CI for the initial (2021) and follow‐up assessment (2024) per group. *p*‐values represent the outcomes of a two‐way ANOVA reflecting the effects of time, group and time × group‐interaction of the outcome.

**FIGURE 3:**
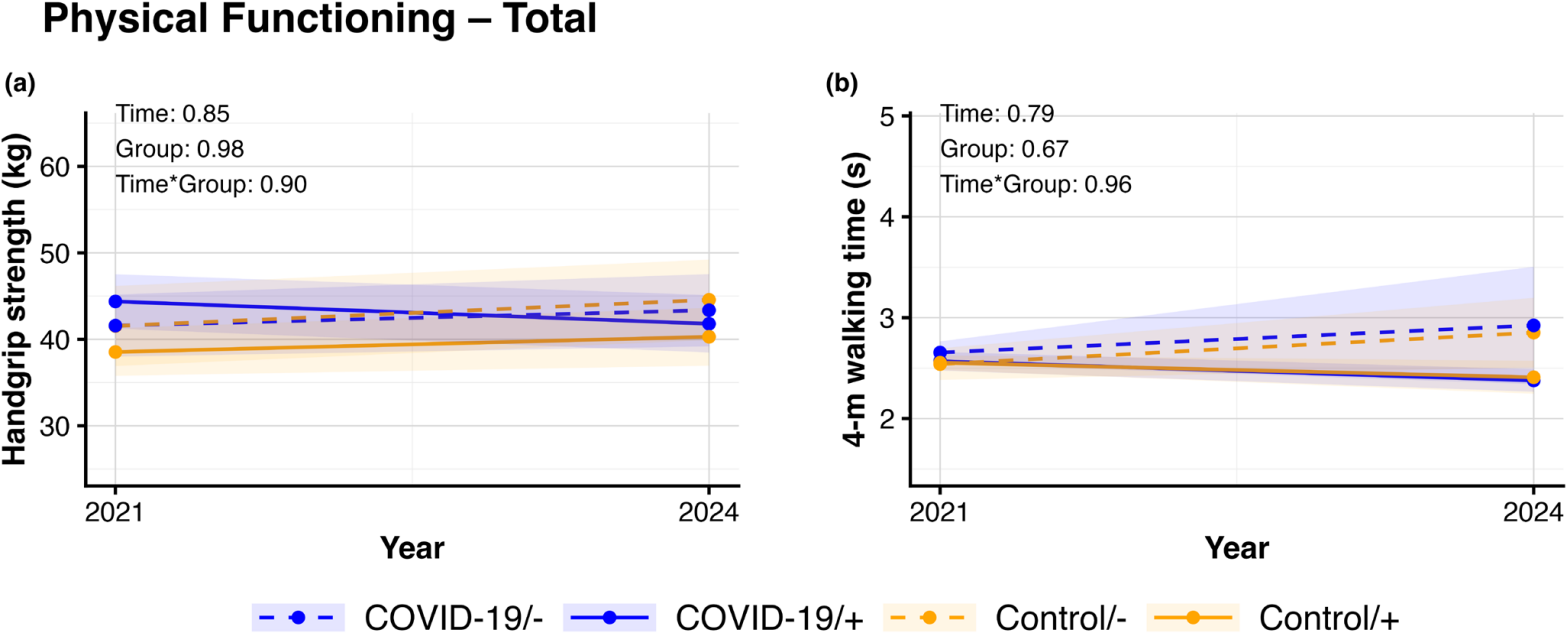
Lines represent data after multiple imputation for handgrip strength (a), and 4‐m walking time (b) which are presented at 6‐months and 3‐years follow‐up following initial COVID‐19 infection (blue) and their age‐ and sex‐matched controls (orange), with both groups divided into those with re‐infection (+, solid lines) or free of re‐infection during follow‐up (−, dashed lines). Lines representing mean and 95% CI for the initial (2021) and follow‐up assessment (2024) per group. *p*‐values represent the outcomes of a two‐way ANOVA reflecting the effects of time, group and time × group‐interaction of the outcome.

**FIGURE 4:**
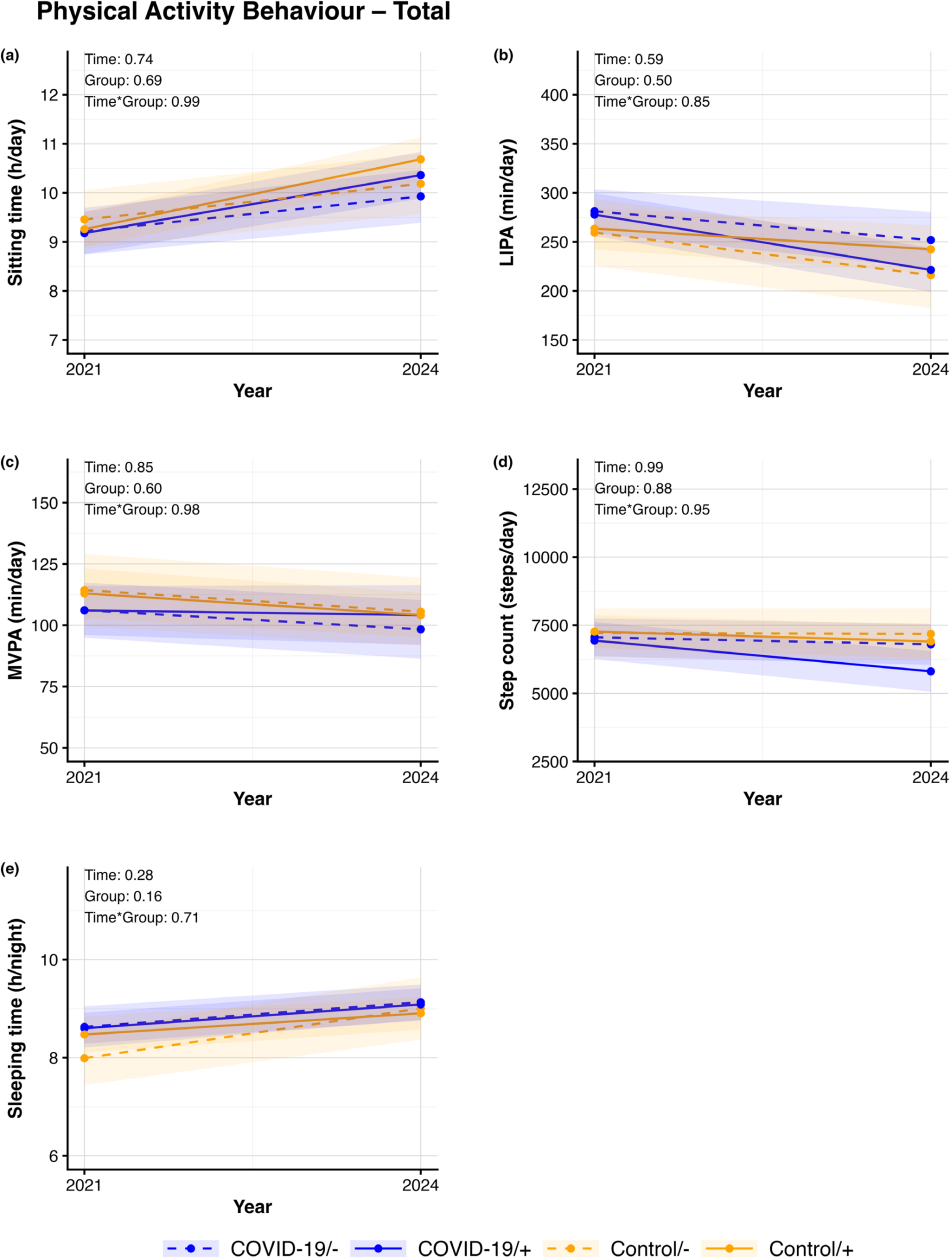
Lines represent data after multiple imputation for physical functioning for sitting time (a), Light intensity physical activity (LIPA, b), Moderate‐to‐vigorous physical activity (MVPA, c), step count (d), and sleeping time (e) which are presented at 6‐months and 3‐years follow‐up following initial COVID‐19 infection (blue) and their age‐ and sex‐matched controls (orange), with both groups divided into those with re‐infection (+, solid lines) or free of re‐infection during follow‐up (−, dashed lines). Lines representing mean and 95% CI for the initial (2021) and follow‐up assessment (2024) per group. *p*‐values represent the outcomes of a two‐way ANOVA reflecting the effects of time, group and time × group‐interaction of the outcome.

We found no changes (all *p*‐values >0.05) in cardiovascular risk factors across follow‐up in the 4 distinct groups (Figure 1). For all cardiovascular risk factors, analyses revealed no significant effect across time, group, or time × group‐interaction. Similarly, no significant changes were found for cardiac biomarkers (all *p*‐values >0.05). No time, group, or time × group‐interaction effects were found in hs‐cTnI and NT‐proBNP across (Figure 2). Lastly, no changes were found across time, group, or time × group‐interaction for any of the markers of physical functioning (Figure 3) or physical activity behaviors (Figure 4) (all *p*‐values >0.05). Additional stratified analyses exploring potential sex‐related differences revealed no significant effects of time, group, and time × group‐interaction for males or females in cardiovascular risk factors, cardiac biomarkers, and physical functioning (all *p*‐values >0.05, Figures [Link], [Link], [Link], [Link], [Link], [Link], [Link], [Link]).

### 3.2 Sensitivity analyses

Baseline characteristics were similar between participants included in the analytical cohort (*n* = 127) versus participants lost to follow‐up (*n* = 75) for cardiovascular risk, cardiac biomarkers, and most, but not all, markers of physical functioning. For example, we found participants from the analytical cohort to demonstrate higher MVPA (109 [89, 137] vs. 95 [80, 120], *p* = 0.009) and step count (7007 [5782, 8871] vs. 6264 [5227, 7687], *p* = 0.018) compared to those lost to follow‐up (Table S2). In a subsequent sensitivity analysis, we repeated our primary analysis using a complete case approach using data from participants who visited for follow‐up measurements, leading to 29 participants in the COVID‐19/− group, 40 in the COVID‐19/+ group, 14 in the Control/− group, and 43 in the Control/+ group. These analyses reinforced the main analyses, showing no time, group, and/or time × group‐interaction effects in any of the parameters (all *p*‐values >0.05, data not shown).

## 4 DISCUSSION

In this study we aimed to explore the long‐term (i.e., 3‐year) changes in cardiovascular risk factors, cardiac biomarkers, physical functioning, and physical activity following non‐hospitalized COVID‐19. First, we found no changes across a 3‐year follow‐up in cardiovascular risk factors, cardiac biomarkers, physical functioning, and physical activity in those with a non‐hospitalized COVID‐19 infection and their age‐ and sex matched controls. Furthermore, these observations were independent of a re‐infection with SARS‐CoV‐2 during follow‐up and similarly present between men and women. Taken together, these observations suggest that an initial SARS‐CoV‐2 infection not requiring hospitalization is not associated with deleterious changes in cardiovascular risk, cardiac biomarkers and physical functioning across 3 years of follow‐up.

In line with our hypothesis and initial observations at 6 months post‐infection (van der Sluijs et al., 2023), we found no change in cardiovascular risk factors, cardiac blood biomarkers, physical functioning, and physical activity across 3 years of follow‐up after the initial COVID‐19 infection. To date, only a few studies have explored the effects of COVID‐19 beyond 1 year. Some studies report altered cardiovascular health in the first weeks or months after COVID‐19 not requiring hospitalization, including mild elevations in cardiac biomarkers, reduced exercise tolerance, or subtle changes in vascular function (Brown et al., 2022; Keefe et al., 2022). Longer follow‐up, including 6‐ or 12‐months, shows a return to normal values, suggesting at least a partial recovery of cardiovascular health over time (Russell et al., 2025; Willems et al., 2022, 2023; Yang et al., 2024). Also, studies with repeated measures up to 18 months post‐COVID‐19 support this notion when examining non‐hospitalized patients for vascular function, inflammation and cardiac biomarkers, and CVD outcomes (Russell et al., 2025; Willems et al., 2022; Willems et al., 2023; Yang et al., 2024). We extend these observations by revealing no change in cardiovascular risk factors and cardiac biomarkers between 6 months and 3 years of follow‐up in non‐hospitalized COVID‐19 patients. This suggests that an initial SARS‐CoV‐2 infection without hospitalization is not associated with long‐term (3‐year) detrimental effects on cardiovascular health.

In understanding our results, it is important to consider that we have included cases with mild COVID‐19 infections only. Requiring hospitalization and/or ICU admission may induce more severe and prolonged cardiovascular effects through impairment of microcirculatory and endothelial function (Poyatos et al., 2024; Sabioni et al., 2023). In support of this, Bruno et al. recently examined arterial stiffness in 2390 individuals ~6 months following COVID‐19, and confirmed a more severe impact of COVID‐19 on arterial stiffness in those requiring ICU admission (Bruno et al., 2025). Moreover, Bruno et al. reported that their observations seem driven by sex differences, as the increased stiffness is primarily driven by observations in women. For this reason, we performed stratified analyses on our data. We could not identify a potential sex difference regarding the long‐term effects of COVID‐19 in non‐hospitalized individuals on our measures of cardiovascular risk and cardiac biomarkers. Severity of disease, but also differences in outcome measures and duration of follow‐up, may explain conflicting results between studies. To better understand the long‐term effects of COVID‐19, we recommend future studies to extend the follow‐up beyond 12 months, but also to assess the role of disease severity, sex and other factors, such as microvascular function, thrombosis, and endothelial function.

A strong point of our study was the evaluation of habitual physical activity and physical functioning, especially given its strong relation with cardiovascular health (Lin et al., 2020). Previously, we observed that the lockdown substantially increased time spent in sedentary behavior (van Bakel et al., 2021), which did not return to pre‐lockdown levels after 3 years of follow‐up (Vloet et al., 2025). COVID‐19 also substantially affects physical activity characteristics in the days and weeks following infection (Gupta et al., 2023; van Bakel et al., 2022). Nonetheless, and in line with our 6‐month observations and our hypothesis, we found no difference in physical functioning and physical activity between non‐hospitalized individuals with COVID‐19 and their age‐ and sex‐matched controls across 3 years of follow‐up. An important consideration is that our participants are recruited from a relatively healthy cohort with participants who nearly all meet the international guidelines of physical activity (van der Sluijs, Bakker, et al., 2025). Given that higher levels of habitual physical activity may protect against long‐term consequences of COVID‐19 (Gomide et al., 2022; Halabchi et al., 2023), the observed long‐term effects of COVID‐19 on our outcomes may have been relatively small. Accordingly, our findings primarily apply to relatively healthy individuals with a low prevalence of pre‐existing comorbidities and cardiovascular risk factors, limiting generalizability to less healthy or less physically active populations.

Another observation from our study is that incident infection(s) did not impact the change in cardiovascular risk factors, cardiac biomarkers, physical functioning, or physical activity between 6 months and 3 years of follow‐up in the non‐hospitalized COVID‐19 participants and those without infection at initial inclusion. To our knowledge, no previous study addressed this topic, while this seems relevant given the relatively high prevalence of COVID‐19 (re‐)infections. An important factor to consider is that the severity of symptoms following a COVID‐19 infection has substantially dropped, including the risk for post‐COVID complaints (Hu et al., 2023). Due to more mild complaints, we may have underestimated the true SARS‐CoV‐2 (re‐)infection rates as we depend on self‐reported re‐infection rather than a PCR‐based confirmation of re‐infection. At the very least, our data suggest that SARS‐CoV‐2 re‐infection, either in those with or without SARS‐CoV‐2 infection in 2020 or 2021, does not alter our outcome parameters across a 3‐year follow‐up.

Our study has several strengths, including the long follow‐up, the within‐subject design, and the comprehensive assessment of cardiovascular health, physical functioning and activity. However, some limitations should be discussed. First, not all participants returned to our laboratory for the 3‐year follow‐up testing. However, the return rate was high (63%), despite such a long follow‐up, and we used multiple imputation techniques to include all participants in our analyses. As multiple imputation under a Missing‐At‐Random assumption may shrink between‐group variability, this approach could bias findings towards the null. However, complete‐case analyses showed similar results, indicating that any imputation‐related bias is likely limited. Second, due to the observational nature of this study, causal relationships could not be established. Although we adjusted for a range of potential confounders, residual and unmeasured confounding cannot be ruled out. Third, our cohort consisted of relatively healthy and physically active adults. Previous work highlighted the benefits of regular physical activity on, for example, body composition and glucose metabolism (van der Sluijs et al., 2025). Therefore, including relatively active participants may attenuate the magnitude of potential long‐term effects and limits generalizability to older or clinically more vulnerable populations. Additionally, detailed information on vaccination timing, vaccine type, and variant exposure was incomplete and partly self‐reported; therefore, these variables were not included in analytical models.

In conclusion, we did not detect evidence that COVID‐19 in non‐hospitalized individuals, who were infected during the first waves of the pandemic in 2020/21, was associated with deleterious changes in cardiovascular risk factors, cardiac biomarkers, physical functioning, or physical activity behavior across 3 years of follow‐up.

## AUTHOR CONTRIBUTIONS

**Janneke I. A. Vloet:** Conceptualization; data curation; formal analysis; investigation; methodology; project administration; resources; software; visualization. **Esmée A. Bakker:** Conceptualization; methodology; supervision. **Koen M. van der Sluijs:** Conceptualization; investigation; methodology; project administration. **Guilherme F. Speretta:** Investigation. **Armando van der Horst:** Resources. **Thijs M. H. Eijsvogels:** Conceptualization; funding acquisition; supervision. **Dick H. J. Thijssen:** Conceptualization; funding acquisition; supervision.

## FUNDING INFORMATION

This project was funded by ZonMW Lifestyle medicine and COVID‐19 SA 2022 (#05550322210002) and by the Dutch Heart Foundation Grant 2020T063. TE was supported by an Established Investigator E‐Dekker grant (#03‐002‐2023‐0036) of the Dutch Heart Foundation.

## CONFLICT OF INTEREST STATEMENT

The authors declare no conflicts of interest.

## ETHICS STATEMENT

This study was conducted in accordance with the Declaration of Helsinki and was approved by the local Ethics committee (2023‐16721). Written informed consent was obtained from all participants prior to their participation in the study.

## Supporting information


Data S1.

**Table S1.** Population characteristics (2021) for participants of the analytical cohort and those lost to follow‐up. For exploring differences between groups for numeric variables, a Wilcoxon Signed Rank test is performed. The chi‐squared test is used for categorical variables. Numbers represent Median [inter quartile range] or *n* (%).

**Figure S1.** Lines represent data after multiple imputation in males for cardiovascular risk factors Body Mass Index (BMI, A), mean arterial pressure (MAP, B), heartrate (HR, C), total cholesterol (D), HDL cholesterol (E), LDL cholesterol (F), triglycerides (G), insulin (H), glucose hexikinase (I), creatinine (J), and CRP (K) which are presented at 6‐months and 3‐years follow‐up following initial COVID‐19 infection (blue) and their age‐ and sex‐matched controls (orange), with both groups divided into those with re‐infection (+, solid lines) or free of re‐infection during follow‐up (−, dashed lines). Lines representing mean and 95% CI for the initial (2021) and follow‐up assessment (2024) per group. *p*‐values represent the outcomes of a two‐way ANOVA reflecting the effects of time, group and time × group‐interaction of the outcome.

**Figure S2.** Lines represent data after multiple imputation in females for cardiovascular risk factors Body Mass Index (BMI, A), mean arterial pressure (MAP, B), heartrate (HR, C), total cholesterol (D), HDL cholesterol (E), LDL cholesterol (F), triglycerides (G), insulin (H), Glucose Hexikinase (I), creatinine (J), and CRP (K) which are presented at 6‐months and 3‐years follow‐up following initial COVID‐19 infection (blue) and their age‐ and sex‐matched controls (orange), with both groups divided into those with re‐infection (+, solid lines) or free of re‐infection during follow‐up (−, dashed lines). Lines representing mean and 95% CI for the initial (2021) and follow‐up assessment (2024) per group. *p*‐values represent the outcomes of a two‐way ANOVA reflecting the effects of time, group and time × group‐interaction of the outcome.

**Figure S3.** Lines represent data after multiple imputation in males for cardiac biomarkers hs‐cTnI (A) and NT‐proBNP (B) which are presented at 6‐months and 3‐years follow‐up following initial COVID‐19 infection (blue) and their age‐ and sex‐matched controls (orange), with both groups divided into those with re‐infection (+, solid lines) or free of re‐infection during follow‐up (−, dashed lines). Lines representing mean and 95% CI for the initial (2021) and follow‐up assessment (2024) per group. *p*‐values represent the outcomes of a two‐way ANOVA reflecting the effects of time, group and time × group‐interaction of the outcome.

**Figure S4.** Lines represent data after multiple imputation in females for cardiac biomarkers hs‐cTnI (A) and NT‐proBNP (B) which are presented at 6‐months and 3‐years follow‐up following initial COVID‐19 infection (blue) and their age‐ and sex‐matched controls (orange), with both groups divided into those with re‐infection (+, solid lines) or free of re‐infection during follow‐up (−, dashed lines). Lines representing mean and 95% CI for the initial (2021) and follow‐up assessment (2024) per group. *p*‐values represent the outcomes of a two‐way ANOVA reflecting the effects of time, group and time × group‐interaction of the outcome.

**Figure S5.** Lines represent data after multiple imputation in males for handgrip strength (A), and 4‐m walking time (B) which are presented at 6‐months and 3‐years follow‐up following initial COVID‐19 infection (blue) and their age‐ and sex‐matched controls (orange), with both groups divided into those with re‐infection (+, solid lines) or free of re‐infection during follow‐up (−, dashed lines). Lines representing mean and 95% CI for the initial (2021) and follow‐up assessment (2024) per group. *p*‐values represent the outcomes of a two‐way ANOVA reflecting the effects of time, group and time × group‐interaction of the outcome.

**Figure S6.** Lines represent data after multiple imputation in females for handgrip strength (A), and 4‐m walking time (B) which are presented at 6‐months and 3‐years follow‐up following initial COVID‐19 infection (blue) and their age‐ and sex‐matched controls (orange), with both groups divided into those with re‐infection (+, solid lines) or free of re‐infection during follow‐up (−, dashed lines). Lines representing mean and 95% CI for the initial (2021) and follow‐up assessment (2024) per group. *p*‐values represent the outcomes of a two‐way ANOVA reflecting the effects of time, group and time × group‐interaction of the outcome.

**Figure S7.** Lines represent data after multiple imputation for physical functioning in males for sitting time (A), Light intensity physical activity (LIPA, B), Moderate‐to‐vigorous physical activity (MVPA, C), step count (D), and sleeping time (E) which are presented at 6‐months and 3‐years follow‐up following initial COVID‐19 infection (blue) and their age‐ and sex‐matched controls (orange), with both groups divided into those with re‐infection (+, solid lines) or free of re‐infection during follow‐up (−, dashed lines). Lines representing mean and 95% CI for the initial (2021) and follow‐up assessment (2024) per group. *p*‐values represent the outcomes of a two‐way ANOVA reflecting the effects of time, group, and time × group‐interaction of the outcome.

**Figure S8.** Lines represent data after multiple imputation for physical functioning in females for sitting time (A), Light intensity physical activity (LIPA, B), Moderate‐to‐vigorous physical activity (MVPA, C), step count (D), and sleeping time (E) which are presented at 6‐months and 3‐years follow‐up following initial COVID‐19 infection (blue) and their age‐ and sex‐matched controls (orange), with both groups divided into those with re‐infection (+, solid lines) or free of re‐infection during follow‐up (−, dashed lines). Lines representing mean and 95% CI for the initial (2021) and follow‐up assessment (2024) per group. *p*‐values represent the outcomes of a two‐way ANOVA reflecting the effects of time, group and time × group‐interaction of the outcome.

Data S2.

## Data Availability

Data will be made available upon reasonable request.
