# Supplementary material for: Changes in cardiovascular health and physical functioning in non‐hospitalized, adult COVID‐19 patients after 3 years of follow‐up
Source: Physiol Rep. 2026 Apr 17;14(8):e70868. doi: 10.14814/phy2.70868 (PMC13090529; doi:10.14814/phy2.70868)
Supplement: Supplementary file 2 — Table S1. Population characteristics (2021) for participants of the analytical cohort and those lost to follow‐up. For exploring differences between groups for numeric variables, a Wilcoxon Signed Rank test is performed. The chi‐squared test is used for categorical variables. Numbers represent Median [inter quartile range] or n (%). [file PHY2-14-e70868-s010.docx]

| 2021 | Analytical cohort  (n = 128) | Lost to follow-up  (n = 74) | p-value |
| --- | --- | --- | --- |
| Age (years) | 58 [54, 65] | 57 [52, 65] | 0.47 |
| Sex (male, %) | 79 (64%) | 37 (51%) | 0.10 |
| Alcohol status (current, %) | 113 (92%) | 62 (86%) | 0.19 |
| Smoking status (never, %) | 71 (58%) | 32 (44%) | 0.33 |
| Vaccination status (vaccinated in 2024, %) | 120 (95%) | NA | NA |
| Cardiovascular risk factors  BMI (kg/m^2^)   - MAP (mmHg) - HR (beats/min) - Total cholesterol (mmol/L) - LDL (mmol/L) - HDL (mmol/L) - Triglycerides (mmol/L) - Insulin (mIU/mL) - Glucose (mmol/L) - Creatinine (μmol/L) - CRP (mg/L)   Cardiac biomarkers   - NT-proBNP (pg/mL) - Hs-cTnI (ng/L) | 24.5 [22.5, 26.4]  98 [93, 108]  57 [53, 64]  5.1 [4.8, 5.6]  3.1 [2.5, 3.6]  1.6 [1.2, 1.8]  1.1 [0.8, 1.4]  4.2 [2.0, 6.8]  4.9 [4.7, 5.2]  81 [72, 90]  4.0 [4.0, 4.0]  8 [5, 12]  4 [3, 7] | 24.3 [22.6, 26.3]  98 [90, 108]  58 [51, 65]  5.3 [4.5, 6.1]  3.3 [2.5, 3.7]  1.6 [1.3, 1.8]  1.0 [0.8, 1.4]  3.9 [2.0, 6.9]  5.0 [4.8, 5.3]  80 [70, 88]  4.0 [4.0, 4.0]  8 [5, 12]  4 [3, 7] | 0.97  0.50  0.88  0.23  0.50  0.43  0.53  0.60  0.40  0.40  0.11  0.68  0.80 |
| Physical functioning  Handgrip strength (kg)  4-m walking speed (km/h)  24-hour physical activity behaviour   - SB (h/day) - MVPA (min/day) - LIPA (min/day) - Step count (steps/day)   Sleep (h/day) | 42 [32, 52]  5.6 [5.2, 6.2]  9.2 [8.2, 10.3]  109 [89, 137]  270 [213, 317]  7007 [5782, 8871]  8.4 [7.3, 9.4] | 37 [31, 48]  5.4 [5.0, 6.1]  9.4 [8.4, 10.4]  95 [80, 120]  242 [205, 330]  6264 [5227, 7687]  8.6 [7.7, 9.3] | 0.11  0.19  0.54  0.009  0.38  0.018  0.32 |

*BMI: Body Mass Index, SB: Sedentary behaviour, MVPA: Moderate-to-Vigorous Physical Activity, LIPA: Light Intensity Physical Activity, MAP: Mean Arterial Pressure, HR: Heart Rate, LDL: Low-Density Lipoprotein, HDL: High-Density Lipoprotein, CRP: C-Reactive Protein, NT-proBNP: N-terminal pro Brain Natriuretic Peptide, Hs-cTnI: High-Sensitivity cardiac Troponin I.*
