# Supplementary figures and images for: Changes in cardiovascular health and physical functioning in non‐hospitalized, adult COVID‐19 patients after 3 years of follow‐up
Source: Physiol Rep. 2026 Apr 17;14(8):e70868. doi: 10.14814/phy2.70868 (PMC13090529; doi:10.14814/phy2.70868)

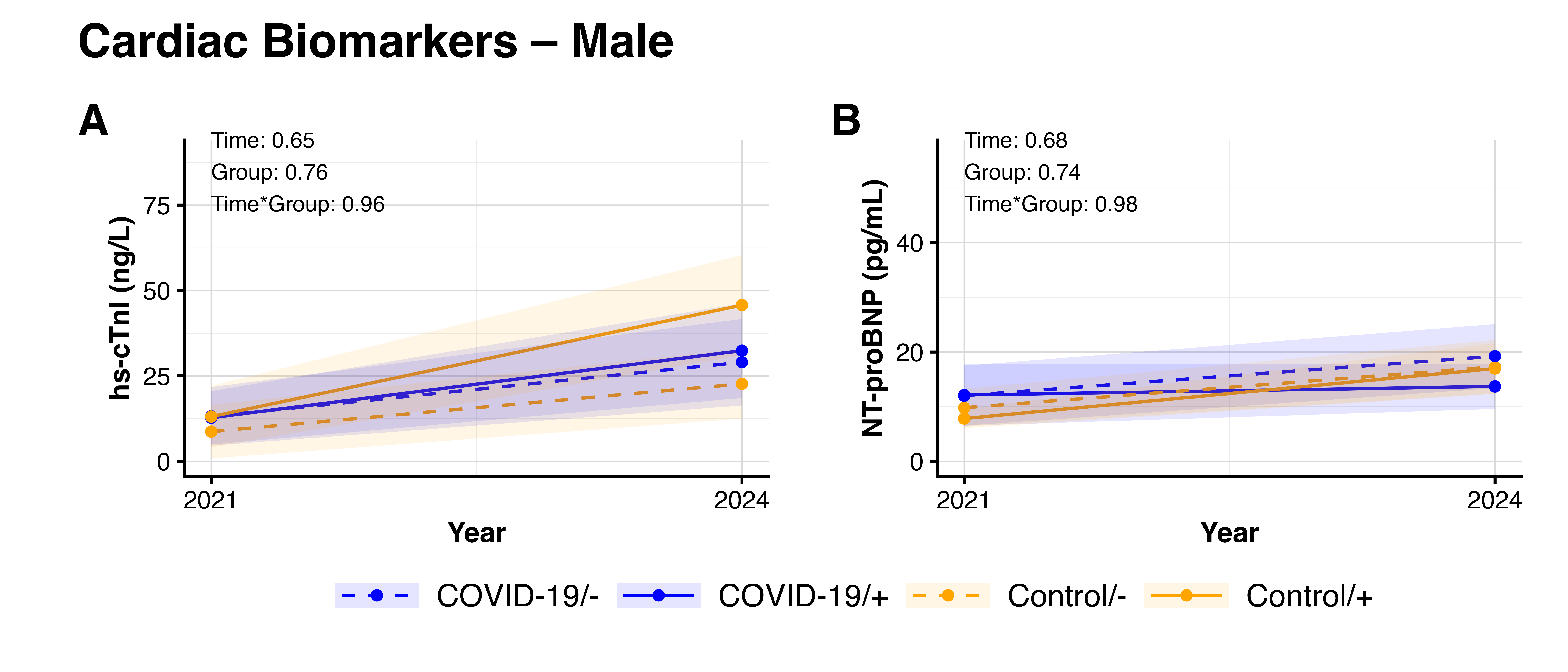

Supplement: Supplementary file 5 — Figure S3. Lines represent data after multiple imputation in males for cardiac biomarkers hs‐cTnI (A) and NT‐proBNP (B) which are presented at 6‐months and 3‐years follow‐up following initial COVID‐19 infection (blue) and their age‐ and sex‐matched controls (orange), with both groups divided into those with re‐infection (+, solid lines) or free of re‐infection during follow‐up (−, dashed lines). Lines representing mean and 95% CI for the initial (2021) and follow‐up assessment (2024) per group. p‐values represent the outcomes of a two‐way ANOVA reflecting the effects of time, group and time × group‐interaction of the outcome. [file PHY2-14-e70868-s006.tif]

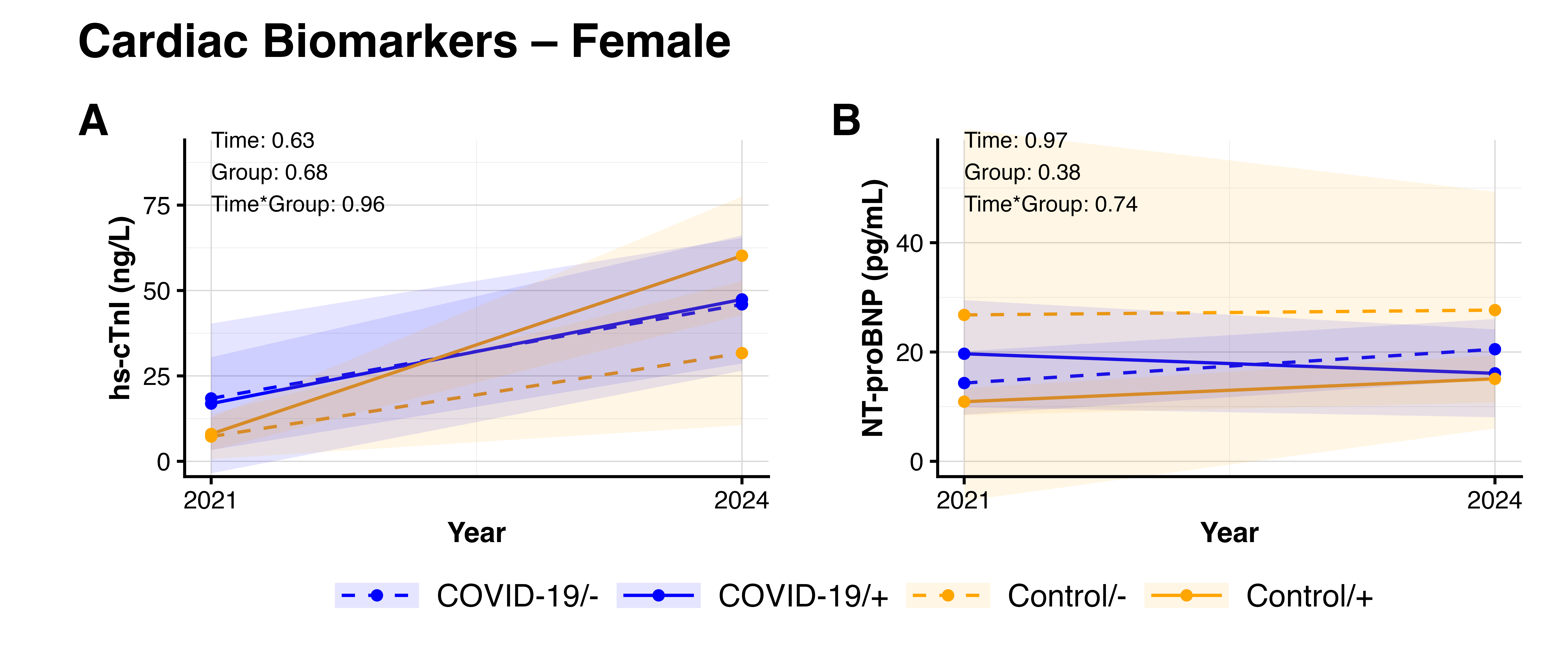

Supplement: Supplementary file 6 — Figure S4. Lines represent data after multiple imputation in females for cardiac biomarkers hs‐cTnI (A) and NT‐proBNP (B) which are presented at 6‐months and 3‐years follow‐up following initial COVID‐19 infection (blue) and their age‐ and sex‐matched controls (orange), with both groups divided into those with re‐infection (+, solid lines) or free of re‐infection during follow‐up (−, dashed lines). Lines representing mean and 95% CI for the initial (2021) and follow‐up assessment (2024) per group. p‐values represent the outcomes of a two‐way ANOVA reflecting the effects of time, group and time × group‐interaction of the outcome. [file PHY2-14-e70868-s011.tif]

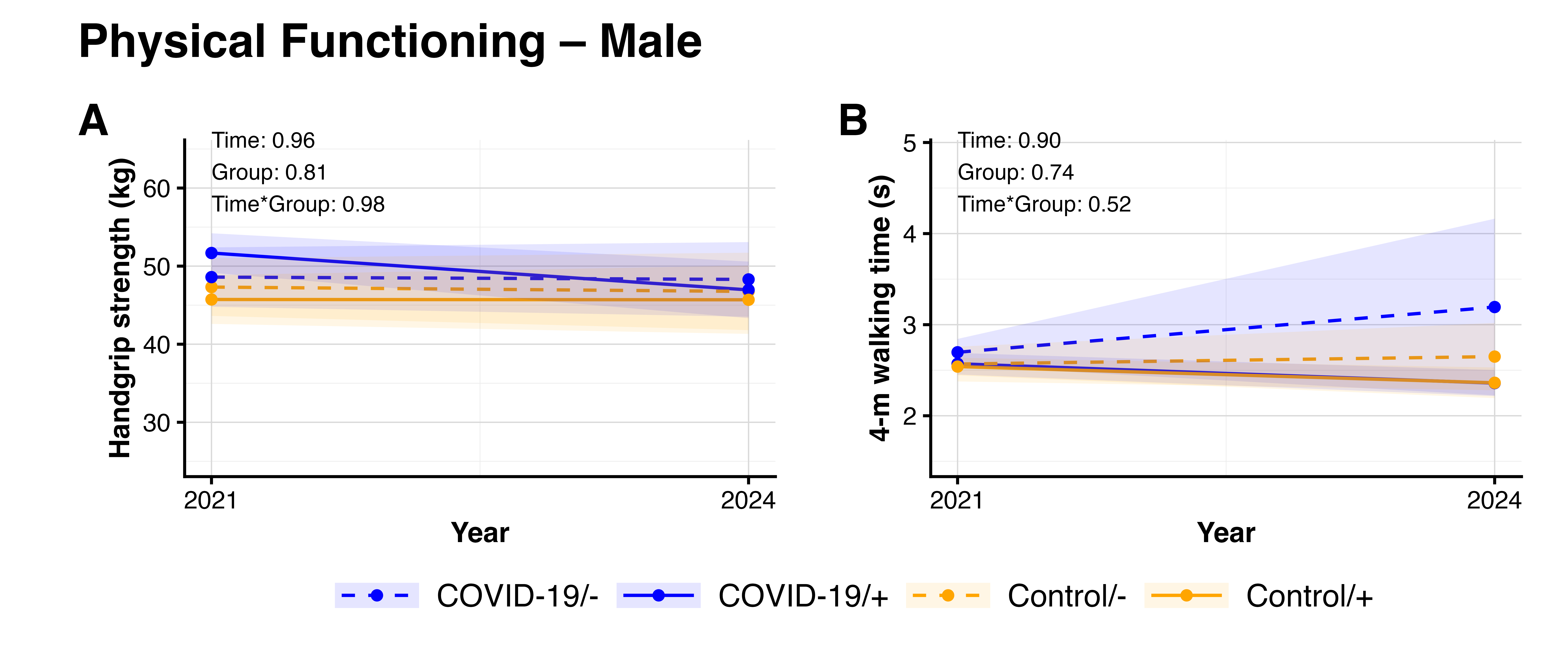

Supplement: Supplementary file 7 — Figure S5. Lines represent data after multiple imputation in males for handgrip strength (A), and 4‐m walking time (B) which are presented at 6‐months and 3‐years follow‐up following initial COVID‐19 infection (blue) and their age‐ and sex‐matched controls (orange), with both groups divided into those with re‐infection (+, solid lines) or free of re‐infection during follow‐up (−, dashed lines). Lines representing mean and 95% CI for the initial (2021) and follow‐up assessment (2024) per group. p‐values represent the outcomes of a two‐way ANOVA reflecting the effects of time, group and time × group‐interaction of the outcome. [file PHY2-14-e70868-s004.tif]

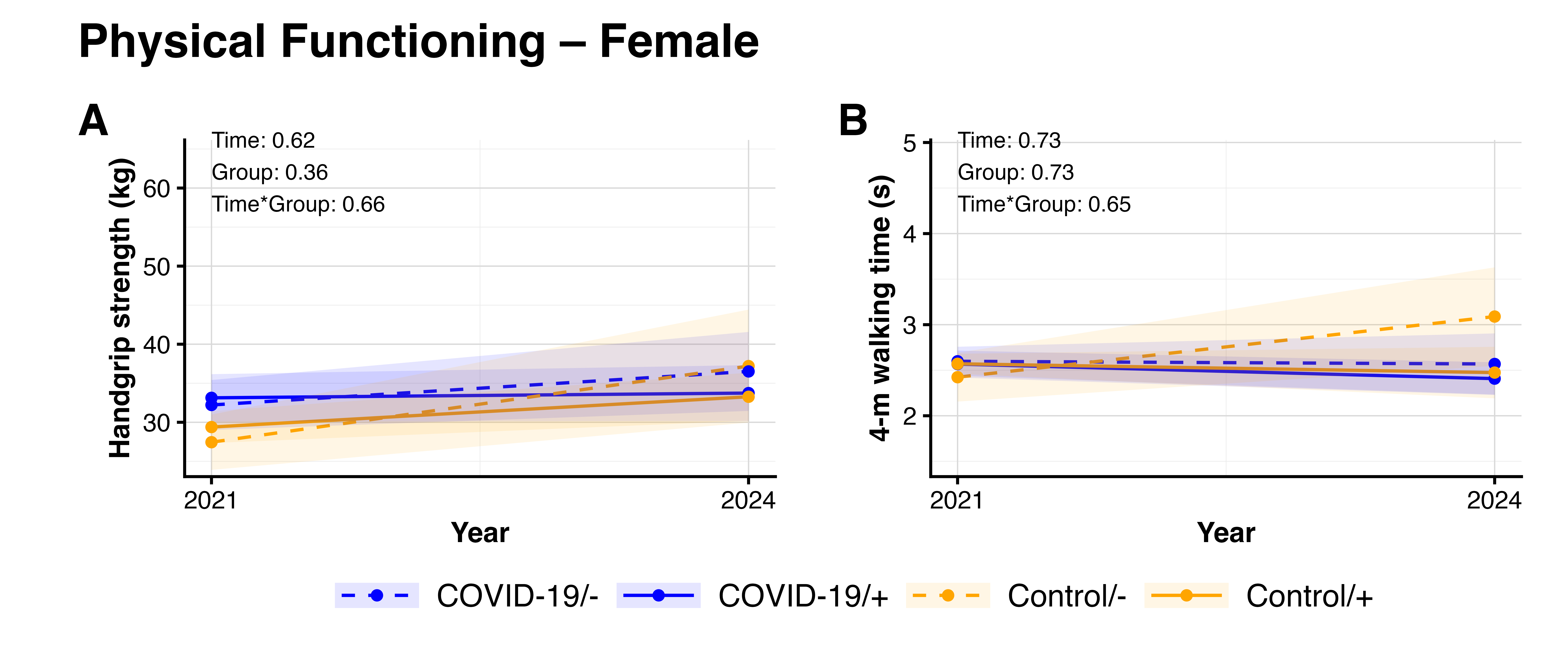

Supplement: Supplementary file 8 — Figure S6. Lines represent data after multiple imputation in females for handgrip strength (A), and 4‐m walking time (B) which are presented at 6‐months and 3‐years follow‐up following initial COVID‐19 infection (blue) and their age‐ and sex‐matched controls (orange), with both groups divided into those with re‐infection (+, solid lines) or free of re‐infection during follow‐up (−, dashed lines). Lines representing mean and 95% CI for the initial (2021) and follow‐up assessment (2024) per group. p‐values represent the outcomes of a two‐way ANOVA reflecting the effects of time, group and time × group‐interaction of the outcome. [file PHY2-14-e70868-s001.tif]
