# Supplementary material for: Changes in cardiovascular health and physical functioning in non‐hospitalized, adult COVID‐19 patients after 3 years of follow‐up
Source: Physiol Rep. 2026 Apr 17;14(8):e70868. doi: 10.14814/phy2.70868 (PMC13090529; doi:10.14814/phy2.70868)
Supplement: Supplementary file 11 — Data S2. [file PHY2-14-e70868-s002.docx]

| Attn:  Prof. Dr. Josephine Adams  Editor-in-Chief  Physiological Reports | | | |  |  | Radboud university medical center  Department of Medical BioSciences  P.O. Box 9101  Route 928  6500 HB Nijmegen  The Netherlands  **Prof. Dr. Dick H.J. Thijssen**  E-mail: Dick.Thijssen@Radboudumc.nl  Telephone: +31 6 11 21 25 34 | |  |  |
| --- | --- | --- | --- | --- | --- | --- | --- | --- | --- |
|  |  |  |  |  |  |  |  | |  |
|  |  |  |  |  |  |  |  | |  |
|  |  |  |  |  |  |  |  | |  |
| Date \| March, 2026; Subject \| Cover Letter | | | | | | | | | |

Dear Prof. Dr. Adams,

Please find enclosed our manuscript, entitled *‘Changes in cardiovascular health and physical functioning in non-hospitalized COVID-19 patients after 3 years of follow-up’*, including the highlighted corrections.

Below, we have again attached the original reviewer reports (#1 and #2), along with our detailed point-by-point responses.

We hope that our manuscript is acceptable for publication in *Physiological Reports*. Thank you for your consideration.

Sincerely yours, on behalf of all authors,

Prof. Dr. Dick Thijssen

Corresponding author

Reviewers' Comments:

Reviewer #1 (Comments to the Author (Required)):

*This longitudinal study examined 3‑year changes in cardiovascular risk factors, cardiac biomarkers, physical functioning, and physical activity in 101 non‑hospitalized adults with SARS‑CoV‑2 infection and 101 age‑ and sex‑matched controls. Participants were assessed at ~6 months (2021) and re‑evaluated ~36 months after infection (2024). Analyses used multiple imputation to address ~37% missing follow‑up data and created four groups based on incident infections during follow‑up (COVID‑19/-, COVID‑19/+, Control/-, Control/+). Outcomes included BMI, blood pressure, lipids, glucose, insulin, creatinine, CRP, hs‑cTnI, NT‑proBNP, handgrip strength, 4‑m gait speed, and 24‑hour accelerometer‑derived activity (sitting time, LIPA, MVPA, step count, sleep). Two‑way repeated measures ANOVA adjusted for age, sex and education tested time, group, and time x group effects. Across all outcomes there were no significant changes or interactions over 3 years (all p>0.05), and sex‑stratified and complete‑case sensitivity analyses confirmed the primary findings. Incident re‑infections and high vaccination rates did not modify results. The authors conclude that non‑hospitalized COVID‑19 during early pandemic waves was not associated with long‑term deleterious changes in cardiovascular health or physical functioning over 3 years, noting limited generalizability to less healthy or hospitalized populations.*

*The introduction clearly identifies long‑term data gap for non-hospitalized COVID‑19 pts and cites conflicting short‑ and mid‑term studies and the authors' own six‑month findings, acknowledging modifiers like disease severity and sex.*

*The introduction (or discussion) should reconcile methodological differences across prior studies (sample size, measurement types, cohort health/fitness, disease severity) that may explain discrepant results. Specifically, the introduction frames the question broadly but does not explicitly state that the cohort is relatively healthy and physically active until later; this is important because the hypothesis may not apply to less healthy or hospitalized populations.*

**Our response:** We agree that this information was now minimally represented in the introduction. We have therefore added the following sentence to highlight the differences across studies (P3, lines 67-68):

“*These discrepant findings may be related to the strong variation in follow-up (3-12 months), disease severity, and population health status, which complicates comparison across studies*.”

*The introduction does not early state that the sample is relatively healthy and physically active, which limits generalizability and should temper the hypothesis.*

**Our response:** We agree that this information was not clearly stated until the end of the manuscript. We therefore have added the following part to the method section of our manuscript, to emphasize the assumed health status of our study cohort (P5, lines 84-85):

“…, a large cohort study including relatively healthy and physically active participants,…”

*There is very little discussion of biological pathways (inflammation resolution, endothelial recovery, viral persistence) that would explain persistence or resolution of effects over three years.*

**Our response:** We appreciate the reviewer’s comment. However, since we did not find differences between groups or changes across time, we believe that discussing potential biological pathways does not fit the main observation within our study. This does not exclude potential persistence of effects of COVID-19 in other (less healthy, less physically active) groups, but this discussion (including potential underlying biological pathways) falls outside the scope of the present study.

*There is insufficient attention to reinfection/vaccination dynamics. Given changing variant virulence, widespread vaccination, and re infections, the introduction could better justify how these factors were expected to influence long term outcomes or the interpretation of a null finding.*

**Our response:** We agree that information about reinfection/vaccination dynamics is important for this topic. However, it remains difficult to gain information on exact reinfection status. Since we only used questionnaires for this, information remains subjective. Additionally, reinfections during later variants were generally milder and vaccination was widespread, potential long-term cardiovascular effects may have been attenuated. We have therefore spent little attention to this subject in our manuscript.

*The hypothesis is partly reasonable given prior six‑month data and some literature showing recovery, but under‑justified because contextual, mechanistic, and statistical considerations are not fully addressed.*

**Our response:** We have added more information to our hypothesis, focussing on the health status of our cohort (P5, Lines 76-79):

“*We hypothesized that, in line with our observations at 6 months post-infection, no longitudinal changes or differences between non-hospitalized individuals with COVID-19 and their controls in cardiovascular risk factors, cardiac biomarkers, physical functioning and physical activity were present in this relatively healthy, non-hospitalized cohort*.”

*Several methodologic questions and clarifications exist: Specify inclusion and exclusion criteria in full, recruitment sources, reasons for nonresponse, and whether baseline health or fitness thresholds were applied.*

**Our response:** We agree that information about inclusion of participants was limited. In- and exclusion criteria were mentioned in the primary study. We have now added this information to the current manuscript as well (P5, Lines 87-92).

“*An inclusion criterion for the COVID-19 group was evidence of a positive polymerase chain reaction (PCR) test for SARS-CoV-2, whereas an exclusion criterion was hospitalization due to the SARS-CoV-2 infection. Age and sex-matched controls were only included if they never had signs, symptoms, or suspicions of a SARS-CoV-2 infection nor a lifetime positive test of any sort for SARS-CoV-2 before study participation. Dutch language proficiency and residency were inclusion criteria for both groups*.”

*Regarding COVID status and reinfection ascertainment, report diagnostic criteria (PCR, antigen, clinical), timing of initial infection, objective confirmation of reinfections versus self-report, and severity scoring for each episode.*

**Our response:** Information about inclusion criteria has been added (see answer on previous comment). Other information about (re-)infections has not been collected.

*Provide a power calculation with assumed effect sizes and minimal clinically important differences for key outcomes.*

**Our response:** We thank the reviewer for pointing out this lack of clarity. We have now added the *a posteriori* power calculation to the methods section, based on the mean arterial pressure (MAP) (P6, Lines 101-106).

***“Sample size***

*As our study has an explorative character, no formal sample size calculation was performed a priory. In the primary study, the Cohen’s D effect size for the mean arterial pressure was 5, with an alpha of 0.05 and beta of 0.2 (power of 0.8). According to G*power (Version 3.1), the (a posteriori) estimated sample size needed was 79 participants in each study arm, so 158 in total. As groups were further subdivided based on COVID-19 status in the present study, these analyses should be interpreted as exploratory.”*

*Exact intervals between infection, baseline (2021) testing, and follow‑up (2024) for each participant and how variable timing was handled should be reported.*

**Our response:** The aim of the primary study was to include participants ~6 months post-infection. The primary study presented that the median time between infection and inclusion was 175 [126-235] days. Follow-up assessment was performed ~30 months after initial assessment. This information has been provided in the methods section of the manuscript (P5, Lines 85-86, 92-93).

*Detail variables used in the imputation model, number of imputations, convergence diagnostics, missingness patterns, and justification for the missing at random assumption should be detailed.*

**Our response:** The methods section already states the following information (P8, Lines 150-152):

“*All mentioned variables present from the assessment in 2021 and 2024 were used to predict missing values in 5 imputed datasets with 20 iterations. Healthy convergence, imputed distribution, and plausibility were verified.”*

We believe this to provide sufficient information on te background and assumptions regarding our multiple imputation.

*List all covariates considered, criteria for inclusion in models, treatment of education, medication use, comorbidities, and whether time‑varying confounders were addressed.*

**Our response:** The methods section already states the following information (P8, Lines 155-156):

“*Level of education, age, and sex were considered as confounders in the analyses.”, which we considered to provide sufficient information on our analyses*.

*If space allows (or possibly as supplement), provide standardized protocols for blood pressure, blood draws, assay methods including limits of detection and inter/intra‑assay coefficients of variation for biomarkers, and calibration procedures for devices. The reviewer appreciates this may not be feasible given the word count restriction - though the paper seems to have space.*

**Our response:** We thank the reviewer for this suggestion. Standardized protocols for blood draws are already present in the current version of the manuscript (P6, Lines 116-119). The biochemical assays follow the manufacturer’s validated procedures.

*Regarding accelerometer processing, report wear‑time algorithms, nonwear rules, sleep/wake diary integration, cutpoints used to define LIPA and MVPA, minimum valid days, and steps algorithm - as space permits.*

**Our response:** We have added more details to the accelerometer processing steps, including minimum wear days. Information on algorithm use was already provided (P7, lines 135-140).

*“A minimum of 4 valid days was used as an inclusion criterion for analysis. Data were extracted via PALbatch (PAL software suite, version 8, PAL technologies) and analyzed using a modified version of the script by Winkler et al. (12, 13) in SAS (Statistical Analysis System, RRID” SCR_008567, version 9.4; SAS Inst., Cary, NC_ to compute daily light intensity (LIPA) and moderate-to-vigorous physical activity (MVPA) duration (min/day), sleeping and sitting time (h/day), and step count (steps/day).”*

*Physical function testing could detail handgrip dynamometer make/model calibration, trial standardization, and gait speed instructions and timing methods.*

**Our response:** The methods section provides the following information, which we expect to provide sufficient information on the physical function tests (Page 7, Lines 128-133):

*“Peak handgrip strength (kg) of the nondominant hand was measured three times, separated by 1-min intervals using the Jamar hydraulic, according to Webb et al. (11). The single highest value was used in the analysis. Gait speed (km/h) was assessed twice over a 4-m stretch with 2-m acceleration and deceleration zones on either side to ensure a stable pace. The fastest try was used in the analysis. This method follows the 4-m gait speed protocol, as described in the Short Physical Performance Battery (11).”*

*Indicate whether assessors were blinded to group status and describe training and interrater reliability.*

**Our response:** Assessors were not blinded to group status, which we now explicitly state in the methods section. All assessments were performed by trained research staff following standardized protocols identical to those used during baseline testing (P8, lines 142-144).

*“Assessors were not blinded to group status. All measurements were conducted by trained staff using standardized protocols identical to the 2021 assessments to ensure consistency and minimize interrater variability.”*

*Statistical model specifications are needed. State full mixed model or ANOVA specifications, covariance structures, checks of model assumptions, multiple testing correction, and rationale for using Control/- as reference.*

We appreciate this request for additional detail. We now specify the full ANOVA model structure (P8, lines 154-157). Because our repeated-measures design involved two timepoints, no explicit covariance structure needed to be modelled. No multiple-testing correction was applied, consistent with the exploratory and hypothesis-driven nature of the study.

*“To compare differences between groups, 2-way repeated measures analysis of Variance (ANOVA) was performed, where Control/- served as a reference group. Given two repeated measurements, no additional covariance structure was required. Level of education, age, and sex were considered as confounders in the analyses.”*

*Fully describe planned sensitivity tests (complete case, alternative imputation, subgroup analyses), thresholds for consistency, and any post hoc analyses.* 
**Our response:** Information about the planned sensitivity tests was provided in the methods section (P8, Lines 158-161).

“*Sensitivity analyses were performed using only the complete cases available within the dataset, as well as sensitivity analyses to assess potential differences between participants who participated in the follow-up measurements and those who did not respond to the invitation*.”

*Vaccination and variant context is needed for interpretive value. As such, provide participants' vaccination timing and type, and, if possible, circulating variant periods to contextualize exposures ---- appreciating data availability and space limitations.*

**Our response:** We appreciate the reviewer’s suggestion and agree that vaccination timing and variant context can provide important interpretive nuance. However, we lacked sufficiently detailed and objective verified data on vaccination timing, vaccine type, and circulating variants for each participant. Reinfection and vaccination were determined via questionnaire only, and variant type was not available. Because these data were incomplete and partly self-reported, we refrained from including them in analytical models to avoid introducing misclassification bias. We now state this explicitly in the discussion (P12, Lines 270-271).

“*Detailed information on vaccination timing, vaccine type, and variant exposure was incomplete and partly self-reported; therefore, these variables were not included in analytical models*.”

*The discussion generally interprets the null findings appropriately, highlighting alignment with the authors' six-month data and other studies showing recovery by 6 to 12 months, and it rightly notes strengths such as the long follow up and comprehensive measures. However, several areas need clearer linkage between results and interpretation as noted below.

The authors underplay limitations that directly affect inference from a null result. The relatively healthy and active cohort is mentioned but not emphasized as a reason the study may lack external validity for older, sicker, or hospitalized populations.*

**Our response:** We thank the reviewer for this important observation. We have strengthened the discussion of external validity by more explicitly emphasizing that our cohort consisted of relatively healthy and physically active adults with a low burden of comorbidities. We now state that this likely reduced the expected magnitude of long-term effects and limits generalizability to older, less healthy, or hospitalized populations. This clarification has been added to the limitations section of the discussion (Page 13, Line 267-270).

“*our cohort consisted of relatively healthy and physically active adults. Previous work highlighted the benefits of regular physical activity on e.g. body composition and glucose metabolism (32). Therefore, including a relatively active group may attenuate the magnitude of potential long-term effects and limits generalizability to older or clinically more vulnerable populations*.”

*The role of statistical power is not addressed, so it remains unclear whether the study could detect small but clinically meaningful changes.*

**Our response:** The original study was based on feasibility within the Nijmegen Exercise Study cohort. Therefore, no a priori power calculation was performed. We have added information about our a posteriori estimated sample size to the manuscript (P6, lines 101-107).

*The handling of missing follow up data is described, but the discussion should more explicitly evaluate how imputation assumptions might bias toward the null.*

**Our response:** We thank the reviewer and agree that this limitation should be described more explicitly. We have now added a statement to the discussion acknowledging that multiple imputation under a Missing-At-Random assumption may attenuate true between-group differences and bias results toward the null. We also note that complete-case sensitivity analyses yielded similar findings, suggesting such bias is likely modest, but cannot be excluded (P13, Lines 262-265).

*“As multiple imputation under a Missing-At-Random assumption may attenuate between-group variability, this approach could contribute to findings towards the null hypothesis. However, complete-case analyses resulted in results with comparable outcomes, suggesting that potential presence of imputation-related bias may be limited.”*

*Causal language should be more cautious given the observational design; statements suggesting no long-term effect would be better framed as no detectable effect in this sample.*

**Our response:** We appreciate the reviewer’s point and have revised statements suggesting causality. We now consistently use phrasing such as “we did not detect evidence for…” throughout our manuscript. These changes are highlighted throughout the manuscript.

*The discussion briefly considers reinfections and vaccination but stops short of integrating how changing variant virulence and vaccine timing plausibly shaped trajectories and could explain null findings.*

**Our response:** Since we collected questionnaire-based data on these topics, we refrained from using these variables in our analyses. We have no definitive information on virulence variant, and therefore this would remain speculative.

*Finally, mechanistic implications are asserted only weakly: the authors should more directly connect their biomarker and function results to possible biological processes such as inflammation resolution, endothelial recovery, or persistent microvascular injury, and identify what specific future measurements would most usefully resolve remaining uncertainty.*
**Our response:** As our analyses did not reveal group-specific alterations, mechanistic explanations remain speculative; therefore, we have chosen to discuss these only briefly.

**Reviewer #2 (Comments to the Author (Required)):**

The authors examined longitudinal changes in cardiovascular risk factors, cardiac biomarkers, physical functioning and physical activity in non-hospitalized individuals with COVID-19 across a 3-year follow-up period. A total of 101 patients with COVID and 101 age- and sex-matched controls were included. Further categorization into 4 groups was done, based on whether recurrent infection occurred. No changes were found in these parameters compared to controls over follow up. The study is important but there are methodological caveats, outlined below.

Comments:
1. How was the number of participants determined? Sample size calculations would be helpful. If they were not done a priori, and the sample size was determined based on feasibility please state so.

**Our response:** *As answered to the first reviewers’ comment, we have now added more information about the sample size calculation to the manuscript (P6, lines 101-106).*

*“As our study has an explorative character, no formal sample size calculation was performed a priory. In the primary study, the Cohen’s D effect size for the outcome measure mean arterial pressure was 5, with an alpha of 0.05 and beta of 0.2 (power of 0.8). According to G*power (version 3.1), the (a posteriori) estimated sample size needed was 79 participants in each study arm, so 158 in total. As groups were further subdivided based on COVID-19 status in the present study, these analyses should be interpreted as exploratory.”*

2. The categorization of patients into COVID/- and COVID/+ patients based on whether they had recurrent infections is problematic. Patients with multiple infections may be more prone to adverse cardiovascular events. The same is true for the control groups. Participants in the control/+ group had infection at some point, so they are no longer controls. The best comparison would be between COVID/-, COVID/+ and control/- for the total duration of follow up.

**Our response:** *We understand this reviewer’s point. However, although participants with incident infection during follow-up no longer represent infection-naïve controls, this subgroup provides relevant information on the effect of late infections. We believe that insight into these groups is valuable and novel. Therefore, we retained the four-group structure.*

3. The timing of recurrent infection matters and should be reported (for example if it happened close to the baseline infection compared to the end of follow up).

**Our response:** *During follow-up, we did not ask for proof of reinfection with SARS-CoV-2, since self-testing became more standard. Therefore, information on timing of recurrent infection was not present and not provided in the manuscript.*

4. For many cardiovascular parameters, for example, mean BP, cholesterol, triglycerides, NT-pro-BNP, the group COVID/- behaves completely opposite of COVID/+, reinforcing the notion that these represent 2 different phenotypes.

**Our response:** *Although patterns differed somewhat between groups, these variations did not reach statistical significance. Accordingly, these non-significant differences between groups should be interpreted with caution and may reflect random variations within small subgroups.*
